# Comparing the Effectiveness of Human Extracted Teeth and Plastic Teeth in Teaching Dental Anatomy

**DOI:** 10.3390/dj13030105

**Published:** 2025-02-27

**Authors:** Noora Helene Thune, Anna Tostrup Kristensen, Amer Sehic, Julie Marie Haabeth Brox, Tor Paaske Utheim, Hugo Lewi Hammer, Qalbi Khan

**Affiliations:** 1 Institute of Oral Biology, Faculty of Dentistry, University of Oslo, 0316 Oslo, Norway; nooraht@student.odont.uio.no (N.H.T.); annatk@student.odont.uio.no (A.T.K.); j.m.h.brox@odont.uio.no (J.M.H.B.); uxutto@ous-hf.no (T.P.U.); qalbi.khan@odont.uio.no (Q.K.); 2 Department of Medical Biochemistry, Oslo University Hospital, 0424 Oslo, Norway; 3 Department of Plastic and Reconstructive Surgery, Oslo University Hospital, 0424 Oslo, Norway; hugoh@oslomet.no; 4 Department of Ophthalmology, Sørlandet Hospital Trust, 4838 Arendal, Norway; 5 Department of Computer Science, Faculty of Technology, Art and Design, Oslo Metropolitan University, 0166 Oslo, Norway; 6 Department of Holsitc Systems, SimulaMet, 0170 Oslo, Norway; 7 Department of Public Health and Sport Sciences, Inland Norway University of Applied Science, 2406 Elverum, Norway

**Keywords:** clinical dentistry, dental education, dentistry, student, tooth identification, tooth morphology

## Abstract

**Objectives**: A thorough knowledge of tooth morphology, encompassing the detailed structural complexities, is essential for the practice of dental hygienists in all aspects of their profession. The aim of this study was to assess the efficacy of two instructional approaches in tooth morphology education, by analyzing the performance of dental hygienist students trained with human extracted teeth compared to those educated with plastic teeth models. **Methods**: This study included two cohorts of undergraduate dental hygienist students: a control group (n = 27) trained using human teeth, and an experimental group (n = 34) trained using plastic teeth models. Each group underwent two consecutive practical exams where they identified all 32 permanent teeth and 8 deciduous molars. Initially, students were tested on the training material that they were assigned (either extracted human teeth or plastic teeth), and, subsequently, they were tested using the alternative material. Both the number and patterns of identification errors were recorded and analyzed. Paired *t*-tests were used to compare error rates between real and plastic teeth for students trained on either plastic or real teeth, unpaired *t*-tests were conducted to assess differences in performance between students trained on plastic versus real teeth when tested on both tooth types, and Fisher’s exact tests were employed to examine variations in error proportions across maxillary and mandibular tooth categories. **Results**: The control group recorded a mean of 6.41 errors per student (total of 173 errors), with three students (11.1%) failing by committing over 12 errors. Their performance improved to a mean of 5.44 errors (total of 147 errors) when tested on plastic teeth, although the improvement was not statistically significant (*p* = 0.20). Conversely, the experimental group demonstrated high accuracy on plastic teeth, with 19 out of 34 students (55.9%) achieving perfect scores and a total of only 50 errors (mean, 1.47). Their performance, however, declined when tested on real teeth, escalating to a total of 354 errors, with 32 students (94.12%) making more errors on real teeth than on plastic, resulting in a significant increase in errors to an average of 10.41 per student (*p* < 0.001). **Conclusions**: This study demonstrates that students perform best when tested on the materials that they initially were trained with, showing that real teeth provide better educational outcomes than plastic models. This advantage underscores the importance of using natural teeth when learning dental anatomy.

## 1. Introduction

Dental hygienists play an important role in promoting oral health and preventing oral diseases [1]. Their responsibilities include conducting oral health examinations, interpreting radiographs, and providing prophylactic care, which underscores their integral role in the dental healthcare team [1,2]. A comprehensive understanding of tooth morphology, including its pertinent structural complexities, distinctive characteristics, and variations across each individual group of teeth is foundational in all facets of dental hygienists’ practice [3]. Knowledge of tooth morphology is crucial for accurate clinical diagnoses and effective patient treatment. Thus, this foundational knowledge is essential for the education and professional practice of dental hygienists [4].

Dental schools have adopted diverse approaches to help students gain proficiency in tooth morphology. These methods include didactic lectures, flipped classroom models, practical courses such as tooth carving and the identification of plastic and extracted human teeth, alongside various e-learning tools [3,5]. Although each approach targets specific learning objectives, they all have limitations affecting their effectiveness [3]. For instance, while traditional two-dimensional images are widely accessible, they fail to capture the spatial complexity of teeth. Similarly, artificial teaching models made from wax or plastic lack the authentic tactile feedback and anatomical variability of human teeth [3,6,7].

The use of extracted human teeth offers significant educational advantages when ethical and health concerns are adequately addressed [5,8,9]. This principle forms the foundation of the established “tooth identification puzzle” methodology, which has become integral to the curriculum at the Faculty of Dentistry, University of Oslo [9]. Through this hands-on approach, students engage actively by observing and handling extracted human teeth, comprising 32 permanent teeth and 8 deciduous molars, alongside a comprehensive morphology compendium containing detailed illustrations and a schematic dentition diagram [9].

Plastic teeth models have widely been used as a teaching modality due to their accessibility and cost-effectiveness [3,7,10], yet their educational value warrants careful consideration. These models serve as effective memory aids by accurately representing the true dimensions of teeth; however, research conducted on medical undergraduate students suggests that, generally, plastic models may be less effective for knowledge acquisition compared to cadaveric specimens [11]. Despite their advantages in standardization and availability, the inherent limitation of plastic models often fails to capture the natural variations and complexities found in real teeth [3,12]. Such limitations raise important considerations regarding the optimal approach to tooth morphology instruction and the methods that lead to superior knowledge acquisition. While both approaches offer valuable insights into tooth morphology, further analysis is needed to identify strategies that effectively enhance students’ understanding and retention of this crucial area of dental education. The aim of the present study was to compare the learning outcomes of extracted human teeth versus plastic teeth models. The null hypothesis is that there is no significant difference in learning outcomes between the two methods.

## 2. Materials and Methods

### 2.1. Teaching Methods and Examination Approaches for Student Cohorts

This study was conducted at the Faculty of Dentistry, University of Oslo, and at the University of Inland, Norway, with the objective of comparing the effectiveness of human extracted teeth versus plastic teeth in dental morphology education. This study involved two cohorts of undergraduate dental hygienist students from 2024: a control group (n = 27) taught using extracted human teeth, and an experimental group (n = 34) instructed with plastic teeth. The age and sociodemographic backgrounds of the participants are largely comparable, primarily because most students fall within the 20 to 25-year-old age range, and sociodemographic variations within Norway are relatively minor. However, there is a notable gender disparity, with females constituting approximately 95% of the student cohort.

**Figure 1:**
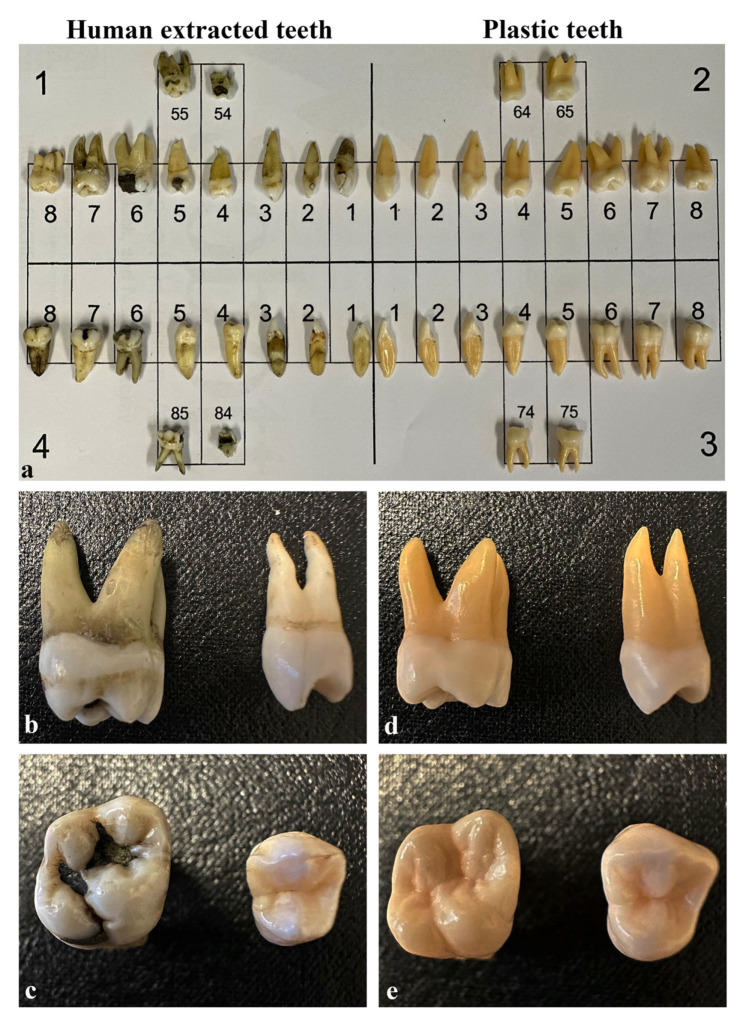
**Examples of Human Extracted and Plastic Teeth:** Panel (**a**) shows a schematic diagram with human extracted teeth (**left**) and plastic teeth (**right**), illustrating significant variations in morphology, size, and color in the real teeth. Panels (**b**,**c**) display the distal and occlusal aspects of a human molar and premolar, respectively. Correspondingly, panels (**d**,**e**) show the distal and occlusal aspects of a plastic molar and premolar.

The teeth used in this study were of undisclosed origin, either provided by affiliated dental offices and sent to the Faculty of Dentistry or collected from the student clinic. All subjects were informed and consented to the use of their teeth for research and educational purposes. According to the Norwegian Regional Committee (REK) for Medical and Health Research Ethics, this use of human teeth falls outside the scope of the Health Research Act and may therefore be conducted without REK approval. Accordingly, the project is conducted responsibly and in compliance with regulations pertaining to informed consent. After the extraction, the teeth were preserved in 70% alcohol for several years in glass jars and thereafter thoroughly dried to ensure the elimination of any organic matter before use. Each set, consisting of 40 teeth, was packaged in a plastic bag and distributed to students either individually or in small groups, based on the availability of materials and student preferences [5]. Prior to their participation, all students were provided with comprehensive information about this research project, including its overall objectives, duration, and the methods and materials involved. The plastic teeth used in this study were supplied by Nissin Dental Products Inc. (Kyoto, Japan) and were distributed in plastic bags, following the same method as for real teeth. Examples of both real and plastic teeth are illustrated in Figure 1.

Both student groups participated in traditional ‘tooth puzzle’ educational methodology, which was previously thoroughly described [5,8]. Briefly outlined, the traditional “tooth puzzle method” required students to correctly identify the full array of 32 permanent teeth and 8 deciduous molars within a schematic dentition diagram according to the FDI World Dental Federation notation system [3]. Both the control and experimental groups undertook a practical examination where they were tasked with identifying 40 teeth. Reference materials were prohibited during the examination, and students were required to achieve a maximum of 12 misidentifications to pass. Each student group completed two consecutive practical identification tests. Initially, they were tested using their assigned training material (extracted or plastic teeth), followed immediately by testing with the alternative material. Moreover, the same instructors graded both tests at both locations, recording both the number and patterns of identification errors, ensuring consistency in pedagogical approach and the documentation of misidentifications.

### 2.2. Statistical Analysis

*T*-tests were applied under the assumption of normality, supported by the central limit theorem given the sample sizes (n = 34 and n = 27), while Fisher’s exact tests, which do not require normality, were used to assess differences in error proportions across maxillary and mandibular tooth categories. For students who received training on plastic teeth during their tooth morphology course, paired *t*-tests were conducted to determine if there was a statistically significant difference (*p* < 0.05) in the number of errors when tested on real versus plastic teeth. Similarly, paired *t*-tests were performed for students who underwent their training using real extracted human teeth to assess potential differences in error rates between the two testing conditions. Furthermore, an unpaired *t*-test was conducted to determine if there was a significant difference in the number of errors for students trained on plastic versus real teeth when tested on plastic teeth. Similarly, an unpaired *t*-test was performed when students were tested on real teeth. Finally, to test if there was any statistically significant difference in the portion of errors for the different tooth categories for maxilla and mandible, exact Fisher tests were used.

## 3. Results

The performance data from the practical “tooth puzzle test” for both cohorts are detailed in Table 1, Table 2 and Table 3. In the control group of 27 students, 2 (7.4%) achieved perfect accuracy with no errors when assessed on the trained material, i.e., real extracted teeth, whereas 12 students (44.4%) had four or fewer errors (Table 1). Collectively, this group recorded 173 errors (mean, 6.41 per student) on the trained material, with three students (11.1%) failing the test by committing more than 12 errors. Interestingly, when tested on the alternative material (plastic teeth), the performance slightly improved, though not significantly (*p* = 0.20), as shown in Table 2. In total, they accumulated 147 errors (mean, 5.44). Only five students (18.5%) increased their error count on the plastic teeth, while the rest maintained or improved their performance (Table 1).

In contrast, the performance of the experimental group, trained on plastic teeth, showed notable differences. When assessed on their trained material, out of 34 students, 19 (55.9%) achieved perfect accuracy with no errors, and the group as a whole accumulated only 50 errors (mean, 1.47) (Table 1 and Table 2). However, their performance on real teeth significantly deteriorated, with the total number of errors escalating to 354. Notably, 32 students (94.12%) recorded more errors on real teeth compared to their initial test on plastic teeth, as shown in Table 1. Testing on real extracted teeth resulted in an average of 10.41 errors per student, a significant (*p* < 0.001) increase from the test on the trained material as detailed in Table 2.

Furthermore, focusing solely on the tests conducted with plastic teeth by both groups (the first column in Table 2), it is demonstrated that students trained on plastic teeth obtained significantly less errors on plastic teeth compared to the students trained on real teeth (*p* < 0.001) (Table 2). The second column, presenting the test on real teeth for both groups, further shows that the students trained on real teeth obtained significantly less errors on real teeth compared to the students trained on plastic teeth (*p* < 0.05), demonstrating superior performance when tested on the trained teeth.

Finally, given that the mean number of errors for the group trained on plastic teeth significantly increased from 1.47 to 10.41 when tested on real teeth (Table 1 and Table 2), we conducted a detailed analysis of the types of errors that increased the most. For this analysis, we categorized the data from the far-right column of Table 1 into five tooth groups for both the maxilla and mandible, presented in Table 3. Our findings revealed significant differences in error rates for both the maxilla (*p* < 0.005) and mandible (*p* < 0.001). Specifically, the most common errors for the maxilla involved canines (39.7%) and molars (20.1%), whereas, for the mandible, the highest error rates were seen with incisors (47.8%), followed by canines (33.8%) (Table 3).

## 4. Discussion

While the use of real extracted teeth has been considered the most important in dental morphology education [9], it has been suggested that incorporating plastic models could supplement learning by enhancing the understanding of complex details and improving overall learning outcomes [13,14]. The primary objective of this study was to compare the learning outcomes in tooth morphology associated with using extracted human teeth versus plastic teeth models. We posited the null hypothesis that no significant differences exist in the learning outcomes derived from these two educational tools. Although students’ perceptions favored real extracted teeth over plastic models [15], and it has been long believed that real extracted teeth offer educational advantages [9], to our knowledge, this is the first study to rigorously evaluate these effects. We accomplished this by comparing two similar groups of students, assessing their performance on both the trained and alternative teaching materials. Consequently, our findings reveal distinct performance patterns between the two teaching modalities. Dental hygienist students, who trained using human teeth, maintained consistent identification accuracy across both test conditions. However, those who were trained using plastic models showed marked disparities in performance when tested on the alternative material, i.e., real teeth. Although they achieved high accuracy with plastic teeth (mean, 1.47 errors), their error rate increased significantly to 10.41 when tested on extracted human teeth. These findings raise fundamental questions regarding the comparative educational outcomes between plastic models and natural teeth in dental education. Plastic models are designed to ideal specifications with uniform shapes and standardized anatomical features across each tooth group. While students often achieve high proficiency with these models, this training may foster a deceptive understanding. When subsequently tasked with examining details and applying knowledge to natural teeth, performance deteriorates significantly, with over 90% demonstrating reduced competency. This suggests that, whereas natural human teeth exhibit anatomical variations that can contribute a more comprehensive understanding, reliance on plastic models may fail to adequately prepare students for teeth’s complexities.

Dental anatomy education represents a complex intersection of theoretical knowledge and practical skill development [3,16]. The approaches employed to facilitate the acquisition of tooth morphology knowledge warrant careful consideration to ensure that they effectively support students’ learning outcomes [7]. Typically, in anatomy courses, educators have long relied on physical models and specimen dissections as primary tools for active learning and the visualization of anatomical structures; however, it has also been consistently demonstrated that incorporating plastic models in anatomy education can enhance students’ ability to visualize and comprehend complex anatomical structures [17]. These results are further supported by Lombardi et al., who showed that students using plastic models achieved significantly higher scores on physiology questions than those engaged in organ dissections [18]. However, taken together, these studies provide compelling evidence for incorporating multiple types of hands-on activities in anatomy laboratory courses, suggesting that a diverse approach to tactile learning experiences may optimize student understanding [18].

Similar trajectories extend to dental anatomy education, where the application of three-dimensional teaching methodologies is essential for achieving optimal knowledge outcomes and clinical success [19,20]. This approach facilitates a comprehensive understanding and learning experience that is crucial for students [5,9]. Overall, evidence on the use of plastic teeth models reveals important details. Regarding students’ perceptions of different instructional modalities in dental morphology education, research indicates that students consider natural extracted teeth to offer the highest educational value, while 3D-printed plastic teeth are rated as the easiest to use [15]. However, in dental morphology education, while plastic models offer a clear visualization of standardized anatomical features, they can oversimplify the natural variations found in actual dental structures that students will encounter in clinical practice [13,15].

A striking finding from this study was the considerable number of misidentifications among students educated in plastic models, who accumulated 354 errors when tested on real teeth. Notably, the identification of mandibular incisors proved particularly challenging, accounting for 47.8% of all errors observed in the mandible and representing the highest error rate among all tooth groups. This confusion likely arises because of their notably symmetrical nature, which makes it difficult when it comes to side determination [21]. These findings are consistent with previous research conducted by Risnes et al. (2018) and Sehic and Khan (2024), which underscore that this is a commonly observed result [5,9]. On the other hand, in the maxilla, the most frequent errors were noted in identifying canines, which accounted for 39.7% of mistakes. The underlying causes for the high error rate with canines remain unclear; however, factors such as the extensive wear on the incisal portion of these teeth and the greater variability in human extracted teeth compared to their plastic counterparts likely contribute significantly to these discrepancies.

Our study also revealed an error pattern specifically among students trained on plastic models when identifying molars in real specimens. Specifically, these students made 41 errors when identifying maxillary molars and 63 errors with mandibular molars, totaling 104 errors in the molar region. This difficulty in identification likely stems from several real-world characteristics absent in plastic models, such as dental restorations, natural variations in both crown and root anatomy, and tooth size. Contributing to this challenge, the human teeth used in this study came from various sources, including dental offices or the student clinic, introducing natural variation not found in standardized plastic models [8]. While students trained on human teeth face these challenges during the course, they are generally more accustomed to the variations found in real specimens, reducing the element of surprise. In contrast, the group instructed using plastic models encountered teeth they had never previously seen, which likely could have influenced their performance in this study’s “tooth puzzle” method to some extent.

The present study also highlights discrepancy between dental hygienist students and dental students receiving an almost identical dental morphology course. The dental hygienists experience significant challenges in mastering tooth identification with natural teeth, with only 7.4% of those trained with natural teeth achieved error-free performance. However, a separate study conducted by Risnes et al. revealed contrasting findings among dental students, where 51.8% demonstrated complete accuracy in their results [9]. Although this may be due to several factors concerning undergraduation, basic science background or perceived relevancy, there is little documentation on this. Future studies should evaluate this further in order to level this discrepancy out.

The present study has some limitations. Firstly, the student cohorts could have been larger and more equally sized, although these discrepancies were statistically corrected. Notably, most participants were female, representing over 90% of the study group, which may limit the generalizability of our results to a more balanced demographic. Additionally, our methods could benefit from being tested on other groups of students, such as dental students, to further validate the results.

## 5. Conclusions and Future Perspectives

The present study demonstrates that students perform better when tested with specimens on which they were initially trained with, a result that is consistent with established expectations. While standardized plastic models serve as a valuable supplementary tool for initial learning, our findings establish that natural human teeth create superior educational outcomes. The experience gained from working with real teeth develops skills that transfers to both real and plastic models. Exposure to natural anatomical variations better prepares students for the complexities and variability encountered in clinical settings, highlighting the importance of incorporating natural teeth in dental anatomy education. We propose that using real extracted human teeth, as opposed to plastic models, may also offer potential benefits for long-term knowledge retention in dental anatomy. However, further research is necessary to comprehensively evaluate this hypothesis. Furthermore, evidence suggests that students utilizing structured digital video-based tools achieve greater learning advancements in dental anatomy [8]. Future research should explore the impact of various digital applications [22], including the potential integration of artificial intelligence [23,24], to further enhance educational outcomes.

## Figures and Tables

**Table 1:** Practical test performance in tooth morphology: Student analysis.

	Students Instructed with *Real* Teeth (Control Group)		Students Instructed with *Plastic* Teeth (Experimental Group)	
Student	No. of Errors Using *Real* Teeth	No. of Errors Using *Plastic* Teeth	No. of Errors Using *Plastic* Teeth	No. of Errors Using *Real* Teeth
**1**	14	12	*0*	*4*
**2**	*14*	*22*	*2*	*9*
**3**	*14*	*20*	*0*	*11*
**4**	10	7	*0*	*2*
**5**	*2*	*5*	*2*	*15*
**6**	4	1	*0*	*4*
**7**	4	0	*2*	*11*
**8**	0	0	*0*	*12*
**9**	4	4	*0*	*10*
**10**	*2*	*6*	*2*	*17*
**11**	*0*	*2*	*4*	*12*
**12**	5	0	*0*	*4*
**13**	4	2	*2*	*9*
**14**	6	0	*0*	*11*
**15**	8	8	*0*	*20*
**16**	10	2	*2*	*11*
**17**	8	8	*0*	*2*
**18**	4	2	*2*	*13*
**19**	4	4	*0*	*14*
**20**	7	0	*6*	*22*
**21**	10	8	*2*	*12*
**22**	10	8	*4*	*18*
**23**	*2*	*6*	*0*	*6*
**24**	5	4	0	0
**25**	4	4	*0*	*6*
**26**	8	8	*6*	*20*
**27**	10	4	*8*	*26*
**28**	-	-	*4*	*13*
**29**	-	-	*2*	*3*
**30**	-	-	*0*	*7*
**31**	-	-	*0*	*18*
**32**	-	-	*0*	*10*
**33**	-	-	*0*	*2*
**34**	-	-	0	0
**Total no. of errors**	173	147	50	354
**Mean**	6.41	5.44	1.47	10.41

The table displays the total number of errors for each group as assessed in both tests. Results from students who exhibited more errors with the alternative material are indicated in *italic*.

**Table 2:** Mean number of errors with standard deviation in parentheses.

Training	Test Plastic	Test Real	*p*-Value
**Plastic**	1.47 (2.11)	10.41 (6.6)	<0.0001
**Real**	5.44 (5.51)	6.41 (4.07)	0.204882
***p*-Value**	0.001233	0.005197	

The first and second rows show the mean number of errors for students trained on plastic and teeth, respectively. The *p*-values compare the expected differences in the respective rows and columns.

**Table 3:** Analysis of error types increasing in the group trained on plastic teeth when tested on real teeth.

	Incisors	Canines	Premolars	Molars	Temporary Molars	*p*-Value
**Maxilla;** **No. of errors (%)**	23 (16.9)	27 (39.7)	26 (19.1)	41 (20.1)	24 (17.6)	0.0044
**Mandible;** **No. of errors (%)**	65 (47.8)	23 (33.8)	37 (27.2)	63 (30.9)	25 (18.4)	<0.0001

The *p*-values test if there are any statistically significant differences in the portion of correct answers for the different categories in both the maxilla and mandible.

## Data Availability

The original contributions presented in this study are included in this article, and further inquiries can be directed to the corresponding author.
